# *HeurAA*: Accurate and Fast Detection of Genetic Variations with a Novel Heuristic Amplicon Aligner Program for Next Generation Sequencing

**DOI:** 10.1371/journal.pone.0054294

**Published:** 2013-01-18

**Authors:** Lőrinc S. Pongor, Ferenc Pintér, István Peták

**Affiliations:** KPS Medical Biotechnology and Healthcare Service Ltd, Budapest, Hungary; National Institutes of Health, United States of America

## Abstract

Next generation sequencing (NGS) of PCR amplicons is a standard approach to detect genetic variations in personalized medicine such as cancer diagnostics. Computer programs used in the NGS community often miss insertions and deletions (indels) that constitute a large part of known human mutations. We have developed *HeurAA,* an open source, heuristic amplicon aligner program. We tested the program on simulated datasets as well as experimental data from multiplex sequencing of 40 amplicons in 12 oncogenes collected on a 454 Genome Sequencer from lung cancer cell lines. We found that *HeurAA* can accurately detect all indels, and is more than an order of magnitude faster than previous programs. *HeurAA* can compare reads and reference sequences up to several thousand base pairs in length, and it can evaluate data from complex mixtures containing reads of different gene-segments from different samples. *HeurAA* is written in C and Perl for Linux operating systems, the code and the documentation are available for research applications at http://sourceforge.net/projects/heuraa/

## Introduction

Analysis of somatic mutations in clinical cancer samples is especially challenging in terms of both collection and computational processing of the data. The experimental difficulties of data collections are due to the small size of tissue sample, to formalin-induced DNA fragmentation as well as to the presence of wild type non-tumor DNA which often dilutes the mutant alleles below detection thresholds. One popular solution is PCR amplification of 100–200 bp long target sequences in cancer related genes followed by sequencing of the PCR products. While Sanger sequencing is generally viewed as the current gold standard, next generation sequencing (NGS) platforms such as the Illumina sequencer, Ion Torrent Personal Genome Machine (ABI) or 454 FLX Genome analyzer (Roche) offer important advantages for amplicon sequencing. For instance, NGS can provide high coverage (1000–10000) of the target sequences, which dramatically increases sensitivity as compared to Sanger sequencing. Therefore NGS can reveal low frequency mutations, which makes the approach an attractive option for diagnostic sequencing. Another advantage of NGS technology for the clinical practice is its ability to deal with parallel sequencing of multiple genes. For instance, re-sequencing of signal transduction genes such as EGFR, KRAS, BRAF etc. is increasingly important approach for personalizing cancer therapies [1]. Recently, researchers at the Massachusetts Hospital proved the clinical usefulness of simultaneous analysis of 12 genes in lung cancer [2]. The 2011 White Paper of the American Society of Clinical Oncology [3] suggested that, independently from the tumor type, all targeted drugs should be registered based on the molecular profile. Therefore there is a strong clinical need for targeted re-sequencing of dozens of genes in each cancer patient. There are several, commercially available multiplex re-sequencing assays in clinical use today (http://www.illumina.com/products/truseq_custom_amplicon.ilmn, http://www3.appliedbiosystems.com/cms/groups/applied_markets_marketing/documents/generaldocuments/cms_094273.pdf). A typical application example is a panel of 40 PCR amplicons taken from 12 genes. Before sequencing, a barcode DNA sequence of 10–12 bp is added to each set of amplicons which enables parallel sequencing of several different amplicons from different patients [4]. We have developed a similar diagnostic panel of 12 cancer genes (Oncompass™ 1.0).

Processing high throughput NGS data for diagnostic purposes has its own challenges. Apart from the obvious needs for accuracy, scalability and reliable patient identification, a data processing pipeline has to be able to handle the widest possible range of mutations. Even though SNPs constitute the majority of somatic mutations listed in the Human Gene Mutation Database [5], insertions and deletions account for about one third of the known mutations. Our lab is specifically interested in an 15 bp deletion within exon19 of the Epidermal Growth Factor Receptor (EGFR). EGFR Exon 19 deletions are known to be sensitizing to EGFR tyrosine kinase inhibitor (TKI) therapy [6], [7]. We identified our first exon 19 mutant non-small cell lung cancer patient with multiplex brain metastasis in 2003 [8]. She was treated with gefitinib which achieved complete remission within months and the patient remained in remission for more than 5 years [7]. In a subsequent study we found complete response in all our exon 19 mutant patients to *EGFR* TKI with 100% response rates in more than 50% of the cases [7].

In a search for reliable and productive data processing alternatives capable of identifying this and similar long deletion mutations in NGS data, we tested several open source data processing tools. In our preliminary analysis, it was disturbing to notice that several open source data processing programs failed to identify the exon 19 mutations and other large deletions and insertions of medical interest, and those that were able to identify them required long processing time. From the technical point of view, this is not entirely surprising. On one hand, exhaustive sequence alignment methods are capable of detecting all mutations, and several vendors promote expensive, dedicated hardware for this purpose. On the other hand, most of the popular, heuristic alignment tools are not necessarily optimized for targeted re-sequencing of amplicon reads since they were developed with the needs of genome analysis in mind. For instance, elegant solutions were developed for efficiently indexing genome sequences [9]–[12]. A remarkable speed increase was achieved by a cache-oblivious, “substitution only” strategy used by the mrsFAST program [13]. However this approach is not designed for detecting indels. So, for the moment, experimenters in need of reliable diagnosis are usually left with time consuming computational methods. This is not reassuring, especially in view of the rapidly growing diagnostic needs [14].

In order to address the above issues we have developed *HeurAA*, an open source data processing program designed for the specific needs of diagnostic re-sequencing of amplicon reads generated by high coverage next generation sequencers. *HeurAA* (heuristic amplicon aligner) uses a combination of exact string matching and exhaustive sequence alignment algorithms which improves accuracy in comparison with other popular, open source aligner programs, especially in the detection of indels. At the same time, a substantial – over one order of magnitude – speed increase is achieved which facilitates the analysis of large datasets on simple desktop computers.

## Results

### Principle

**Figure 1:**
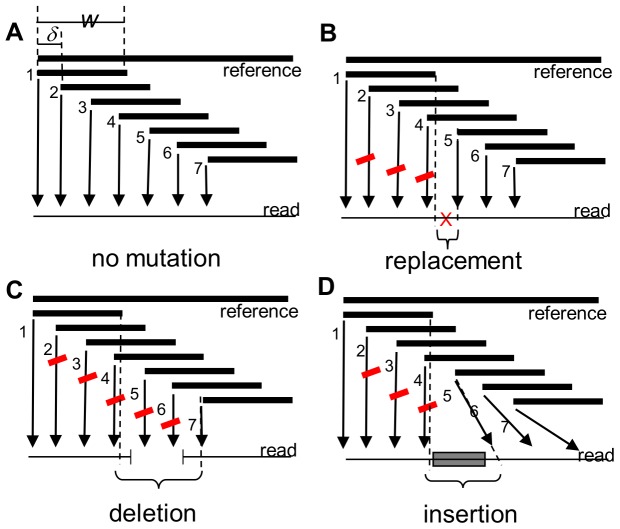
Mapping replacements, deletions and insertions with words tiling the reference sequence. Words of length *w* are positioned at *delta* bps from each other. The non-matching words (red) allow for the identification of the polymorphic region, i.e. the segment in which the mutation is found.

**Figure 2:**
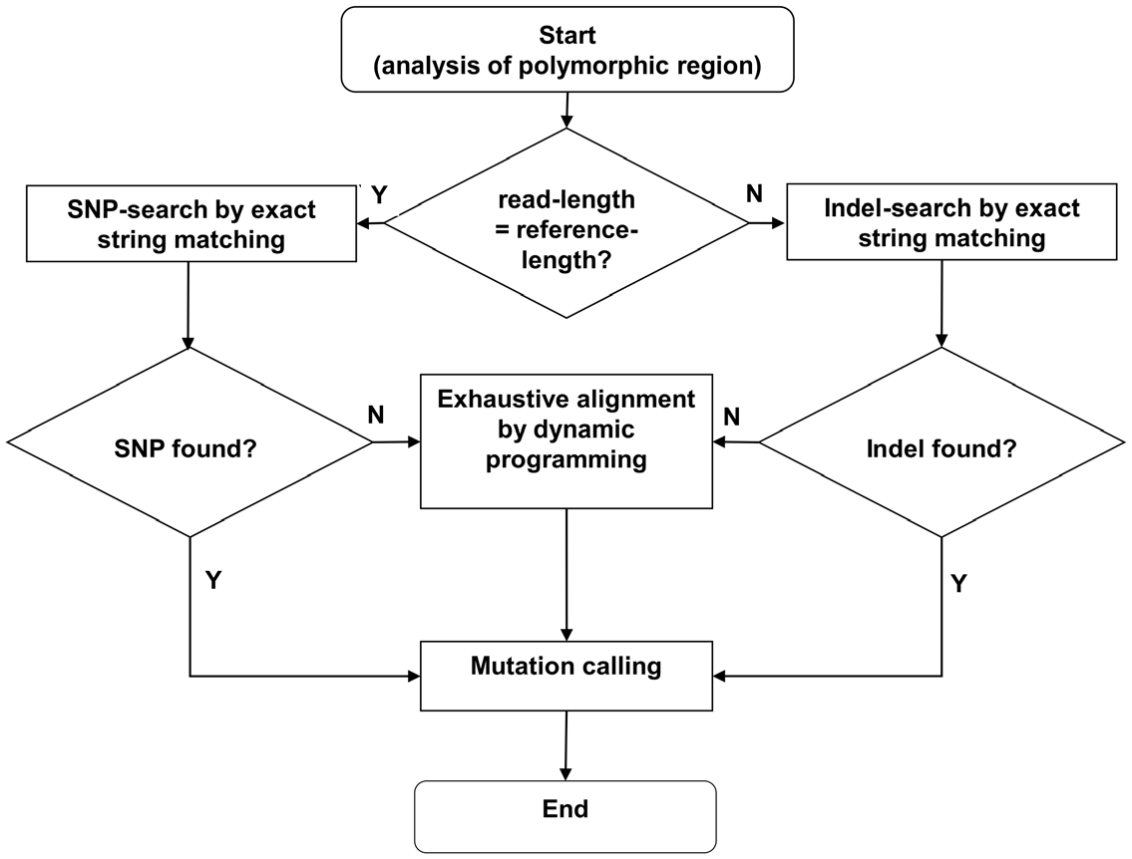
Analysis of the polymorphic region (schematic flowchart). Each polymorphic region within a read is analyzed according to this general procedure. Dynamic programming refers to the use of the Needleman Wunsch or the Smith Waterman algorithm Mutation calling refers to converting the results of the previous steps into standard mutation description format (http://www.hgvs.org/mutnomen/recs-DNA.html) [20].

Given a reference sequence and a set of amplicon reads, *HeurAA* will use a regularly spaced sub-word tiling of the reference sequence and an exact string matching algorithm in order to a) filter out malformed reads and b) to locate potential regions in which mutations are found. A schematic outline of the subword tiling is shown in **Figure 1**. The non-matching sub-words are used to identify the so-called polymorphic region which contains the mutations with respect to the reference sequence. After this step of search space reduction, *HeurAA* will use an exact, character-by-character comparison to locate single substitutions, insertions and deletions (**Figure 2**). If more than one of these are found within a certain distance, *HeurAA* will consider the mutation as “complex” and use the Needleman-Wunsch [15] or and the Smith-Waterman algorithm [16] to align the corresponding regions of the read and the reference sequence. Definitions and details of the algorithm are described in the Methods section. *HeurAA*’s accuracy is guaranteed by the fact that exact string matching is used for all mutations except complex ones which are in turn analyzed by a rigorous alignment algorithm. Speed is secured by the fact that *strstr,* a very fast string matching function of GNU C library (http://www.gnu.org/software/libc/) is used for sequence comparison, and the time-consuming operations are restricted to very short sequence segments.

Highly repetitive reference sequences may cause trivial problems when string recognition is carried out with a substring search algorithm, such as *strstr,* that only reports the first occurrence of a substring. *HeurAA* circumvents this problem by tagging repetitive words in the reference sequence before the analysis, and then successively looking for each of their occurrences in the read.

### Run Times

Current protocols recommend the sequencing of a few hundred bp long segments around a mutation of medical interest, so first we consider run times at a constant reference length covered by a read of the same length. In principle, the run time of the algorithm at is then expected to depend a) on the w word-size, b) on the *delta* tiling shift, c) on the repetitiveness of the reference sequence and d) on the nature of the polymorphisms found (namely, complex polymorphisms require dynamic programming alignment) Typical run times for a non-repetitive exon amplicon at w = 15 and *delta* = 5 are shown in **Table 1**.

**Table 1:** Typical run times for non-repetitive reads*.

	Operation	Time
1	Index construction	1∼ msecs
2	Sub-word-identification (strstr calls):	6.2 sec
3 or 4	Identification of one simple mutations,	1.7 sec
	Identification of one complex mutation(Needleman-Wunsch)	16.3 sec

* Calculated for 1,000,000 copies of a 225 bp segment taken from exon 20 of the human EGFR gene (see Exon 20 reference sequence in Supplementary Materials **S1**). Simple mutations are randomly placed single substitutions or deletions of arbitrary size. Complex mutations were generated by placing two random substitutions plus a randomly placed 4 bp deletion within a window of 15 nucleotides.

**Figure 3:**
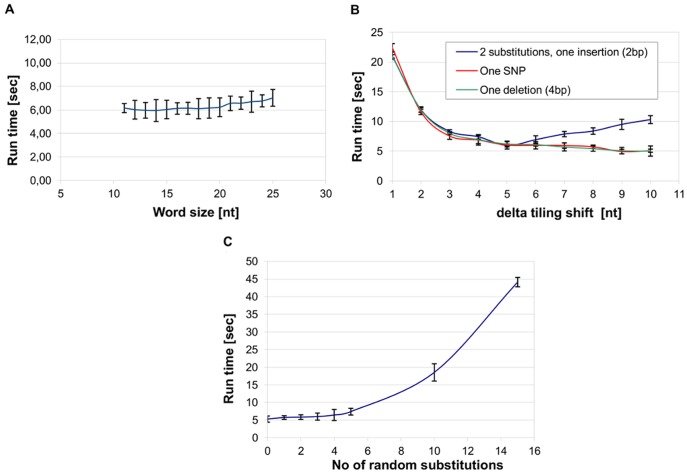
Dependance of runtime on various program parameters. **A)** Dependence of the running time on the value of *w* word size. **B)** Dependence of the running time on the value of *delta* tiling shift. Run times given for 500,000 copies of the Exon_20 reference sequence (see Section 4.1 as well as Supplementary Materials **S1**). Error bars indicate +/−3 standard deviations calculated from 10 repetitions. *w* = 15 and *delta* = 5 were used unless otherwise indicated. We note that irrespective of the choice of parameters shown in the figure, *HeurAA* found the mutations in all cases. C**)**“No of random substitutions” indicate the number of substitutions randomly introduced into the same read.

When tested on simulated sequences, we found that the run-time apparently did not appreciably depend on the word-size (**Figure 3A**). The setting of *delta* was found to be more crucial (**Figure 3B**), as it directly influences a) the number of *strstr* calls as well as b) the length of the polymorphic region within which detailed comparisons are made (**Figure 3B**). More precisely, at larger *delta* values there will be less *strstr* calls, but at the same time more character-to-character matching steps will be necessary for identifying the polymorphism. This latter effect is especially pronounced in the case of complex mutations that are analyzed by dynamic programming; in such cases we see an “optimal” delta value (see curve “2 substitutions, one insertion” in **Figure 3B**). We found that *delta* = 5 and w = 15 are appropriate choices for ∼200 bp exon reads, as well as for simulated reads ranging from 100 to 5000 bp in length, so these parameters were chosen as the default values of the program. It is noted that accuracy did not apparently depend on the values of *delta* or w, all mutations were found at all parameter settings in the experiments shown in **Figure 3**. Regarding the increasing trend in **Figure 3C**, we note that, as described in the Methods section, simulated mutations were randomly distributed along the length of the read. Consequently, there was a growing probability that two mutations would fall within a window of 2w+*delta* = 35, causing *HeurAA* to use the Needleman Wunsch algorithm which explains in turn the increasing trend of the run-time at higher mutation values. We also note that multiple substitutions and deletions are rare among the disease mutations found *in vivo*.

As future sequencing protocols may produce substantially longer reads than current techniques, we also estimated the dependence of run times on the length of longer reads and reference sequences. We found, that the dependence is linear, i.e. by using the same word length and tiling shift values, increasing the reference/read length ten times will increase run times ten times (data not shown).

### Accuracy and Comparison with Other Programs

The performance and accuracy of *HeurAA* was explored in comparison with several popular mapping tools, such as Needleman-Wunsch (NW) [15], *BWA* [12] (version 0.6.1), *Bowtie* [11] (version 0.12.7), Bowtie 2 [10] (2.0.0-beta5), *mrsFAST* [13] (version 2.1.0.3) *and* MOSAIK version 1.1.0014). For the comparison we used both simulated and experimental datasets. The simulated datasets were generated by placing random mutations into the same reference sequence, i.e the mutations were randomly placed at any position of the sequence. The reference sequence was a 225 bp segment of the EGFR gene which is known to harbor cancer related mutations. The data summarized in **Table 2** show that Needleman-Wunsch, BWA, Bowtie2 and *HeurAA* could detect 100 percent of the mutations in simulated datasets. In the experimental datasets, the genetic mutations (SNPs or deletions) were at the same position of the reference sequence, but random mutations (sequencing errors) could occur at any position of the sequence. In the experimental datasets, Needleman-Wunsch and *HeurAA* could detect all mutations, followed by BWA (97.8% for a single SNP and 99.8% for a single deletion) and Bowtie2 (30.3% for a single SNP and 88.48% for a single deletion).

**Table 2:** Accuracy and speed of mutation mapping.

	Simulated datasets^a)^								Experimental datasets(b)			
	SNP(1)		SNP (2)		Deletion (3 bp)		Deletion (15 bp)		Cell line H1975 (c)(one SNP)		Cell line HCC827(d)(Deletion 15 bp)	
Algorithm:	Time (sec)	%	Time (sec)	%	Time(sec)	%	Time(sec)	%	Time (sec)	No of hits(% correct)	Time (sec)	No of hits(% correct)
Needleman-Wunsch [15]	132600	100	135600	100	136600	100	129800	100	204000	1000000 (100)	99000	1000000 (100)
BWA [12]	1264	100	1306	100	1234	100	1276	100	1641	998142 (99.8)	913	997564 (97.88)
Bowtie [11]	88	100	92	100	154	0	152	0	144	325812 (32.49)	95	29700 (0)
Bowtie2 (beta) [10]	146	100	148	100	144	100	146	100	379	988372 (98.84)	212	985512 (97.14)
mrsFAST [13]	14	100	14	100	16	0	14	0	15	640727 (64)	16	68280 (0)
Mosaik(e)	3860	100	4086	100	3824	0	3870	0	4926	254718 (25.4)	1426	83784 (0)
HeurAA(this work)	7,1	100	7,5	100	7,6	100	6,9	100	23	1000000 (100)	25	1000000 (100)

(a) 1,000,000 sequences of 225 bp, containing the indicated number of different, randomly placed substitutions, insertions or deletions generated by the MSBAR program of th EMBOSS package [25]. The reference sequence was a part of exon 20 of the Human Epidermal Growth Factor Receptor gene (NT_033968/ENSG00000146648) exon 20, 4838312 - 4838535 bp, sequence in Supplementary Materials **S1**).

(b) Data collection was carried out by PCR amplicon sequencing on a Roche 454 sequencer as recommended for the 454 platform^ (^ http://454.com/applications/targeted-resequencing/index.asp ^).^ Reads containing complete sequence identifiers and longer than 80 bp were analyzed further.

(c) The samples were taken from a culture of H1975 cells carrying a a 128C>T mutation in exon 20 of the EGFR gene [26] (COSMIC [24] ID: 6240). The reference sequence was the same as in (a). 1,000,000 reads containing complete sequence identifier and longer than 80 bp were analyzed.

(d) The samples, taken from a culture of HCC827 cells carrying a 137delGGAATTAAGAGAAGCA mutation in exon 19 of the EGFR gene [27] (COSMIC ID: 6223, sequence in Supplementary Materials **S1**). The reference sequence was a segment of the EGFR gene(NT_033968/ENSG00000146648) exon 19, 4831698 - 4831757 bp segment, sequence in Supplementary Materials **S1**). 1,000,000 reads containing complete sequence identifier and longer than 80 bp were analyzed. Part of the dataset is deposited with the Supplementary Materials **S1**.

(e) http://bioinformatics.bc.edu/marthlab/Mosaik.

As the detection of large deletions seem to be problematic for several popular programs [14], we analyzed simulated datasets corresponding to known genetic variations that contain large deletions/insertions in the same region (**Table 3** **)**. The octapeptide repeat region of human major prion protein was chosen as a prototype example of a repetitive protein [17]. An approximately 200 bp region was chosen that contains five 24 bp near-perfect repeats, with the structure of R1-R2-R2-R3-R4, where R1 is a nonapeptide repeat, R2 is present in two identical copies. A natural variant of this region contains a 24 bp deletion between R1 and R2 (no. 2 in **Table 3**), while disease-linked mutations include several single or multiple insertions of 24 bp repeats [18], [19]. The sequence of the insertion often correspond to variants of the R2 repeat, which gives rise to structures such as R1-R2-R2’-R2-R2’R3-R4 (no 3 in **Table 3**) where R2’ differs from R2 by a single nucleotide substitution (sequences given in Supplementary Materials **S1**). Again, random SNPs are correctly located by all the programs, but the deletions are detected only by Needleman-Wunsch, BWA, Bowtie2 and *HeurAA*.

**Table 3:** Comparison of typical run-times(a) and hit accuracy measured on the prion octapeptide repeat region.

	Random SNP(1)(b)		Deletion of 24 bp(c)		Insertion of 48 bp(c)	
	Time (sec)	% found	Time (sec)	found	Time (sec)	found
*NW* [15]	153228	100	153228	+	153228	+
*BWA* [12]	1270	100	1298	+	1304	+
*Bowtie* [11]	190	100	24	−	22	−
*Bowtie2* [10]	346	100	428	+	446	+
*mrsFAST* [13]	16.4	100	14.8	−	14.8	−
*Mosaik* (b)	5430	100	5912	−	6490	−
*HeurAA*	8.12	100	9.24	+	9.42	+

(a) The times were measured for 1,000,000 amplicons harboring different randomly placed substitutions.

(b) SNP(1) sequences were produced by placing 1 random substitution in to the repeat region using the MSBAR program of the EMBOSS package [25]. The times were measured for 1,000,000 randomly generated read copies.

(c) The times were measured for 1,000,000 copies of the same sequence.

(b) http://bioinformatics.bc.edu/marthlab/Mosaik, version 1.1.0014.

In terms of running times, *HeurAA* seems to be well over an order of magnitude faster than the next fastest program that is capable of detecting large indels. We note that the running time of *HeurAA* also includes multiplexing as well as mutation calling, i.e. generation of a standard format of the mutation found [20], and this step is not part of the other programs included in the comparison. On the other hand, mutation calling and multiplexing are crucial for the practice of clinical laboratories.

Multiplexing – the simultaneous testing of different gene-segments in different patients in the same sequencing run – is a crucial feature required for large scale analysis of genetic mutations which drastically improves the throughput of a diagnostics laboratory. Multiplex sequencing is made possible by ligating sample specific oligonucleotide barcode(s) to the amplicons of every paitent. For the analysis, *HeurAA* uses an initial demultiplexing step wherein the program associates the sequencing reads to a sample using a user-defined multiplex file that contains the sample-to-barcode associations. *HeurAA* currently allows for the analysis of up to thousands of gene-segments in up to around hundred of patients. The number of patient samples and reference sequences is limited only by the RAM size. In our hands, 10 samples with 26 reference sequences could be routinely analyzed on laptop with 3Gb RAM.

**Figure 4:**
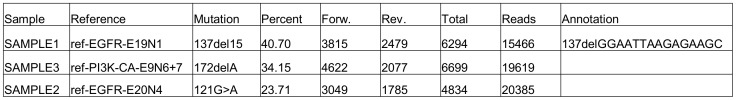
*HeurAA* output. Sample: Name of the sample; Reference: name of the reference sequence; Mutation: Mutation found (mutations with >5 nucleotides are replaced with the length of the mutation); Percent: Percentage of mutation in all sequences identified with specified sample an reference; Forw: Number of mutations found in forward sequences; Rev: Mutations found in reverse complement sequences; Mut no.: Total number of mutations found; Reads: Total reads found for specified sample/reference pair; Annotation: For mutations longer than 5 nucleotides), *HeurAA* prints out the entire mutation here.

A typical output example is shown in **Figure 4**.

## Discussion

*HeurAA* is a novel algorithm developed for a specific clinical application area, targeted resequencing of multiple genes in multiple patients, using a combination of exact string matching and exhaustive dynamic programming algorithms. This is a noteworthy difference between *HeurAA* and the other alignment tools that in most cases use heuristic approximations developed for genome analysis. The underlying philosophies are also different: many genome aligners us the “seed and extend” approach borrowed from BLAST [21], i.e. roughly speaking, they locate the regions of highest similarity in the first step and extend it in both directions in the second step. This is an adequate approach for finding regions of high similarity in a background of variable sequence segments. However, targeted resequencing is a different task where the goal is to locate regions of relatively small differences in a background of completely identical regions. *HeurAA*’s approach is designed to take advantage of this particular situation. Namely, *HeurAA* first excludes the identical sequence segments from the further analysis, using exact string matching (strstr calls), and zooms on the polymorphic region. In the second step, the polymorphic region is further analyzed to find the exact mutations. So this is rather a “zoom and explore” approach, as opposed to the “seed and extend” philosophy underlying many heuristic genome aligners.

In comparison to the popular open source software tools currently used by the NGS community, *HeurAA* is considerably – by over one order of magnitude – faster when applied to amplicon analysis, and also more accurate in detecting indels (see Tables 2, 3). *HeurAA* can detect indels practically without size limits provided the reads contain a part (say, at least 30 bps) of the reference sequence that is sufficient for accurate identification. The speed of *HeurAA* is based on a combination of several factors: a) A simple and memory-resident index structure is used, that is fast to build and can be stored in memory, even for a large number of reference sequences. b) This index structure allows one to use *strstr*, a simple string-matching algorithm that has a highly efficient, assembly-based implementation in GNU C library (http://www.gnu.org/software/libc/). The current version *HeurAA* is designed to run on ordinary desktop or laptop computers. We expect that using faster implementations and dedicated architectures will bring substantial further improvement since the algorithm can be easily parallelized. Nevertheless, we estimate that even the current version of the program can process the output of several sequencers in real time, which is sufficient to analyze several hundred samples per day without a dedicated hardware infrastructure.

The next version of *HeurAA* to be released in the near future will include options for command line use, parallelization as well as the use of the SAM format in order to increase compatibility with high throughput workflows.

Accuracy is of prime importance in diagnostics settings so we believe that *HeurAA* and other, dedicated software tools will help high throughput targeted re-sequencing research projects as well as foster the clinical use of NGS diagnostics in the personalized treatment of cancer. It is often argued that the continuous improvements in whole genome sequencing might ultimately erode the *raison d’etre* of targeted re-sequencing approaches. We are however convinced that targeted re-sequencing has inherent advantages as opposed to whole genome sequencing, such as a significant reduction of costs and efforts and an increased focusing on regions of interest, which is important both for diagnosis as well as for regulatory agencies [22]. In conclusion, we believe that targeted re-sequencing is likely to remain the most economical option for many years to come in various application fields, so development of dedicated software tools will continue to be a worthwhile effort.

## Data and Methods

### Experimental Datasets

Experimental datasets were obtained from cancer cell lines carrying mutations in exon 19 or exon 20 of the human EGFR receptor gene. Data collection was carried out by PCR amplicon sequencing on a Roche 454 sequencer as recommended for the 454 platform (http://454.com/applications/targeted-resequencing/index.asp), using the Oncompass panel of 40 PCR amplicons taken from 12 genes (EGFR, KRAS, NRAS, HRAS, HER-2, BRAF, KIT, PDGFR, AKT, MET, ALK, PIK3CA) (OncompassTM 1.0; KPS) developed for targeted re-sequencing of cancer diagnosis samples. Raw datasets of approximately 300,000 reads were collected for each cell line. Two datasets containing mutations found in the COSMIC database (http://www.sanger.ac.uk/genetics/CGP/cosmic/) [23], [24] were used in further comparisons. In order to benchmark all algorithms on an equal footing, raw datasets were pre-filtered so that only reads containing valid molecular identifiers and longer than 80 bp were used in the analysis. Dataset 1 (8610 reads) contained a 225 bp segment corresponding to exon 20 of human EGFR receptor, with a 128C>T mutation (COSMIC ID: 6240) while Dataset 2 (5867 reads) contained a 286 bp segment corresponding to exon 19 of human EGFR receptor, with a 137delGGAATTAAGAGAAGCA mutation (COSMIC ID: 6223). These data were used for the calculations summarized in Table 2.

### Simulated Datasets

Simulated datasets were generated with the MSBAR program of the EMBOSS package [25], placing an appropriate number of random mutations (replacements, single or block indels) into a 225 bp sequence taken from exon 20 of the human EGFR gene reference (sequence shown Supplementary Materials **S1.**).

Another simulated dataset was built using a 200 bp segment of the octapeptide repeat region of human major prion protein protein [17]. Here we first generated the known indel variations, and then added a random SNP to each of them. This resulted in dtasets of 1 million reads (see description at Table 3). The sequences are shown in Supplementary Materials **S1**).

### The *HeurAA* Algorithm

The input of the algorithm is a reference nucleotide sequence, and a set of nucleotide sequence reads. Both are typically 100–500 bp in length.

Definition 1: The segment of a read sequence that contains mutations is called a *mutated segment*. The segment of the reference sequence that is mapped to the mutated segment is called a *polymorphic region*.

*HeurAA* uses a sparse overlapping tiling of the reference string (**Figure 1**) to build a memory-resident index list. The index is built from two lists, which contain the sequences of sub-words of length *w* starting at sequence positions 1, *delta*, 2*delta*, 3*delta*… respectively both from the forward and the reverse complement strand of the reference sequence. If the last sub-word is shorter than *w*, its sequence is added to the previous sub-word. The resulting list is short since a reference sequence of length *l* will give rise to a maximum of *[l-w+1]* sub-words of size *w*, and this maximum is reached when *delta* = 1.

The *polymorphic region* will be mapped out by the non-matching sub-words in such a way that a mutation will map between the end of the last matching word preceding it, and the beginning of the first matching word following the mutated position(s) (**Figure 1B-1D**). The figure also shows that the regions flanking the mutation cannot be longer than *delta*, which sets an upper limit to the length of the polymorphic region found by the algorithm.

**Figure 5:**
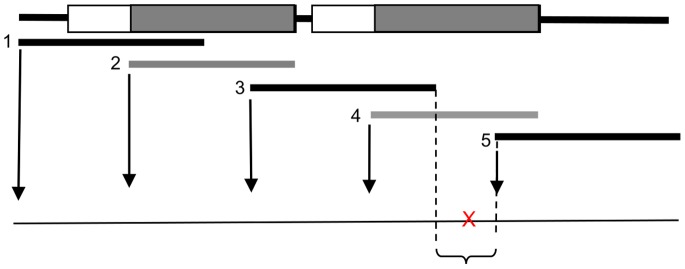
Repeats in the reference sequence. The repeats are indicated by boxes. Note that the *strstr* function detects only the first occurrences of the words so a mutation occurring within a region covered only by the subsequent occurrences (grey) will not be noticed unless the algorithm specifically looks for them.

Note that if all sub-words of the reference are different, a simple exact string matching function – one indicating the presence of a sub-word by the starting position of the first occurrence - would be sufficient to locate the polymorphic region. Reference sequences containing internal (**Figure 5**) repeats pose problems in this respect since a mutation in the second internal repeat would not be detected once the first internal repeat is found (**Figure 4**). Second, a mutation may lead to word repetition in the read, so a word present only once within the reference will appear at two different locations within the read. Consequently, all positions of the repeating words have to be recorded.

**Figure 6:**
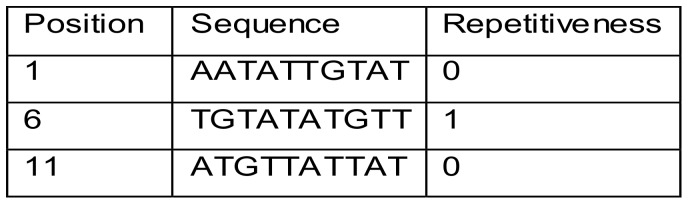
Logical structure of Index file (example). In this example the sub-word found in position 6 is also found in at least one other location of the reference sequence (see example in Figure 5).

In practice, *HeurAAA* translates the reference sequence into a word index list that contains the following data for each subword: the starting position within the reference sequence, the sequence and a flag variable indicating whether or not the word is found at multiple positions (**Figure 6**).

Exon sequences which are the predominant targets in diagnostic sequencing are typically non-repetitive. This means that the index will only contain unique words with no flag indicating repetitivity. On the other extreme, a (hypothetical) homopolymeric reference sequence (say, 100 ‘A’s) would contain the same subword at every position of the index, and each would be flagged as repetitive.

For the analysis of the reads, *HeurAA* uses the *strstr* function, an exact string matching algorithm that has highly efficient assembly language implementations, notably in the GNU C Library (such as the current versions of GLIBC). This function returns the starting position of the first occurrence of a word and a null pointer for words not found. For repeating words, *strstr* is applied in a recursive fashion. Very highly repetitive sequences (e.g. reference sequences with dominant homopolymeric runs) require longer run times – we note that such sequences are not frequent targets in medical diagnosis applications.

After locating the polymorphic region, *HeurAA* determines the exact position of the mutations.

Definition 2: The term *simple mutation* refers to cases where the mutated segment contains only a single substitution or a single indel (indels can be single or block insertions/deletions). Otherwise the mutation is called a *complex mutation*.

*HeurAA* employs different search strategies for these two categories. The algorithm will first attempt to locate simple mutations using a character-by-character comparison of the two sequence segments (i.e. the polymorphic region within the reference sequence and the mutated segment within the read). If only one simple mutation is found, its location and type is reported. However, if there is a further mismatch within a window of *w* flanking a simple mutation on either side, the mutation will be considered “complex”, and the two sequence segments will be compared with a dynamic programming algorithm. The Needleman-Wunsch algorithm [15] is used for mutations located within the interior of the reads, and the Smith-Waterman algorithm [16] is used for mutations located at the 5′ or 3′ termini of the reads. Since a simple mutation is located within a window of *delta* the length of a polymorphic region containing a complex mutation consisting of two adjacent substitutions will be 2*w*+*delta* For longer deletions and insertions the region will become correspondingly longer. We note that for the *w* and *delta* values found useful for practical applications, the values of 2*w*+*delta* values are around or below 50 bp, so the time required for the dynamic programming step is not excessive (also see below).

We note that the location of the polymorphic region and the analysis of simple mutations such as substitutions and (block) indels are carried out by exact string matching which is accurate by definition. Complex polymorphisms in turn are characterized by dynamic programming which ensures optimal identification but the alignment pattern will depend on parameters such as gap insertion and gap extension penalties.

### Algorithm Outline

#### Preprocessing

Reading in and checking the input files containing a) patient IDs and associated molecular identifier (MID) sequences in tab-delimited format b) reference sequences, c) MID sequences in concatenated FASTA format and d) sequence reads, both in concatenated FASTA format. The user can select single end (5′) or paired end (5' and 3') barcoding.

### Analysis

#### Index file construction

*HeurAA* builds the index file (Figure 6) based on the *w* word-length and *delta* tiling shift values (Figure 1A).

#### Read filtering

The algorithm will retain those reads that contain a) an intact MID sequence and b) at least predefined number of words in common with the reference sequence (default is 5). At the end of this step, each retained read is characterized by the position(s) of the matching words found.

#### Location of the polymorphic region and identification of simple mutations

*HeurAA* will locate the polymorphic regions defined as the sequence segment between the end of the last matching word preceding, and the beginning of the first word following the mutated position(s). The polymorphic regions are first compared using character-by-character comparison with the corresponding region of the reference sequence. This procedure will allow an accurate identification of point mutations (replacements, indels) as well as contiguous, single deletions or insertions.

#### Analysis of complex mutations and truncated reads

Polymorphic regions that contain more than one simple mutation are deemed “complex” and are further analyzed by the Needleman-Wunsch algorithm [15]. Truncated reads are identified with the Smith-Waterman algorithm [16].

#### Output file generation

The output of this phase is a tab-delimited list of the mutations for each read.

#### Postprocessing

Counting the frequency of individual mutations, preparation of a tabular output (**Figure 4**).

The analysis phase is carried out by the core *HeurAA* program which is written in the C language. The Preprocessing and postprocessing steps are carried out in a wrapper program written in PERL. The source code of the programs, input and output file examples as well as further explanations are deposited at http://hydra.icgeb.trieste.it/~pongor/HeurAA/.

### Parameter Settings and ther Programs Used for Comparison

The performance and accuracy of *HeurAA* was explored in comparison with several popular mapping tools using the following parameter settings:

- Needleman-Wunsch (NW) [15]: The NW algorithm was used as implemented in the EMBOSS program package^3^; matches: 8, mismatches: -2, gapopen: -7, gapextend: -1.
- *BWA* [12] (version 0.6.1): Error threshold, *n = *2/3/4/6 (for different runs); number of mappings: *N* (indicating “all”). Maximum number of locations to be reported for each read, *n = *300,000,000. In addition, we also benchmarked BWA with the default setting that report a unique map location. The BWA-SW algorithm recommended for Roche 454 reads was used. We note that SW option was necessary for BWA to detect long deletions.
- *Bowtie* [11] (version 0.12.7): Error threshold, *v = *2/3 (for different runs); number of mappings: *a* (indicating “all”);
- *Bowtie2* [10] (2.0.0-beta5): default parameters.
- *mrsFAST* [13] (version 2.1.0.3): Number of mappings for any read, *n = *0 (all mappings to be returned); error threshold, *e = *2/3/4/6 (for different runs).
- *MOSAIK* (http://bioinformatics.bc.edu/marthlab/Mosaik, version 1.1.0014), parameters: hash size: 17, mismatch number 4
- *HeurAA* (this work): Word length *w = *15, *delta* = 5. Minimum threshold for shared sub-words = 5

## Supporting Information

File S1 **Reference sequences. Reference sequences used for accuracy and speed comparison.**
(DOCX)

## References

[1] Petak I, Schwab R, Orfi L, Kopper L, Keri G (2010) Integrating molecular diagnostics into anticancer drug discovery. Nat Rev Drug Discov 9: 523–535. 20531274 10.1038/nrd3135

[2] Sequist LV, Heist RS, Shaw AT, Fidias P, Rosovsky R, et al (2011) Implementing multiplexed genotyping of non-small-cell lung cancers into routine clinical practice. Ann Oncol 22: 2616–2624. 22071650 10.1093/annonc/mdr489 PMC3493130

[3] Kris NG, Meropol NJ, Winer EP, editors (2011) ASCO’s Blueprint for Transforming Clinical and Translational Cancer Research, November 2011. 1 ed. Alexandria, VA: American Society of Clinical Oncology. 29 p. 10.1200/JCO.2011.40.1125 22215747

[4] Alkan C, Kidd JM, Marques-Bonet T, Aksay G, Antonacci F, et al (2009) Personalized copy number and segmental duplication maps using next-generation sequencing. Nat Genet 41: 1061–1067. 19718026 10.1038/ng.437 PMC2875196

[5] Stenson PD, Ball EV, Howells K, Phillips AD, Mort M, et al (2009) The Human Gene Mutation Database: providing a comprehensive central mutation database for molecular diagnostics and personalized genomics. Hum Genomics 4: 69–72. 20038494 10.1186/1479-7364-4-2-69 PMC3525207

[6] Jackman DM, Yeap BY, Sequist LV, Lindeman N, Holmes AJ, et al (2006) Exon 19 deletion mutations of epidermal growth factor receptor are associated with prolonged survival in non-small cell lung cancer patients treated with gefitinib or erlotinib. Clin Cancer Res 12: 3908–3914. 16818686 10.1158/1078-0432.CCR-06-0462

[7] Pinter F, Papay J, Almasi A, Sapi Z, Szabo E, et al (2008) Epidermal growth factor receptor (EGFR) high gene copy number and activating mutations in lung adenocarcinomas are not consistently accompanied by positivity for EGFR protein by standard immunohistochemistry. Journal of Molecular Diagnostics 10: 160–168. 18258923 10.2353/jmoldx.2008.070125 PMC2259471

[8] Schwab R, Pinter F, Moldavy J, Papay J, Strausz J, et al (2005) Modern treatment of lung cancer: case 1. Amplification and mutation of the epidermal growth factor receptor in metastatic lung cancer with remission from gefitinib. J Clin Oncol 23: 7736–7738. 16234532 10.1200/JCO.2005.02.4760

[9] Ferragina P, Manzini G (2000) An Experimental Study of an Opportunistic Index; Redondo Beach (CA) USA. IEEE. 390–398.

[10] Langmead B, Salzberg SL (2012) Fast gapped-read alignment with Bowtie 2. Nat Methods 9: 357–359. 22388286 10.1038/nmeth.1923 PMC3322381

[11] Langmead B, Trapnell C, Pop M, Salzberg SL (2009) Ultrafast and memory-efficient alignment of short DNA sequences to the human genome. Genome Biol 10: R25. 19261174 10.1186/gb-2009-10-3-r25 PMC2690996

[12] Li H, Durbin R (2009) Fast and accurate short read alignment with Burrows-Wheeler transform. Bioinformatics 25: 1754–1760. 19451168 10.1093/bioinformatics/btp324 PMC2705234

[13] Hach F, Hormozdiari F, Alkan C, Birol I, Eichler EE, et al (2010) mrsFAST: a cache-oblivious algorithm for short-read mapping. Nat Methods 7: 576–577. 20676076 10.1038/nmeth0810-576 PMC3115707

[14] Oliver GR (2012) Considerations for clinical read alignment and mutational profiling using next-generation sequencing. F1000 Research 1: 1–7. 24627757 10.12688/f1000research.1-2.v1 PMC3945748

[15] Needleman SB, Wunsch CD (1970) A general method applicable to the search for similarities in the amino acid sequence of two proteins. J Mol Biol 48: 443–453. 5420325 10.1016/0022-2836(70)90057-4

[16] Smith TF, Waterman MS (1981) Identification of common molecular subsequences. J Mol Biol 147: 195–197. 7265238 10.1016/0022-2836(81)90087-5

[17] Kretzschmar HA, Stowring LE, Westaway D, Stubblebine WH, Prusiner SB, et al (1986) Molecular cloning of a human prion protein cDNA. DNA 5: 315–324. 3755672 10.1089/dna.1986.5.315

[18] Croes EA, Theuns J, Houwing-Duistermaat JJ, Dermaut B, Sleegers K, et al (2004) Octapeptide repeat insertions in the prion protein gene and early onset dementia. J Neurol Neurosurg Psychiatry 75: 1166–1170. 15258222 10.1136/jnnp.2003.020198 PMC1739180

[19] Goldfarb LG, Brown P, Little BW, Cervenakova L, Kenney K, et al (1993) A new (two-repeat) octapeptide coding insert mutation in Creutzfeldt-Jakob disease. Neurology 43: 2392–2394. 8232966 10.1212/wnl.43.11.2392

[20] den Dunnen JT, Antonarakis SE (2000) Mutation nomenclature extensions and suggestions to describe complex mutations: a discussion. Hum Mutat 15: 7–12. 10612815 10.1002/(SICI)1098-1004(200001)15:1<7::AID-HUMU4>3.0.CO;2-N

[21] Altschul SF, Gish W, Miller W, Myers EW, Lipman DJ (1990) Basic local alignment search tool. J Mol Biol 215: 403–410. 2231712 10.1016/S0022-2836(05)80360-2

[22] Mertes F, Elsharawy A, Sauer S, van Helvoort JM, van der Zaag PJ, et al (2011) Targeted enrichment of genomic DNA regions for next-generation sequencing. Brief Funct Genomics 10: 374–386. 22121152 10.1093/bfgp/elr033 PMC3245553

[23] Forbes SA, Bindal N, Bamford S, Cole C, Kok CY, et al (2011) COSMIC: mining complete cancer genomes in the Catalogue of Somatic Mutations in Cancer. Nucleic Acids Res 39: D945–950. 20952405 10.1093/nar/gkq929 PMC3013785

[24] Forbes SA, Tang G, Bindal N, Bamford S, Dawson E, et al (2010) COSMIC (the Catalogue of Somatic Mutations in Cancer): a resource to investigate acquired mutations in human cancer. Nucleic Acids Res 38: D652–657. 19906727 10.1093/nar/gkp995 PMC2808858

[25] Rice P, Longden I, Bleasby A (2000) EMBOSS: the European Molecular Biology Open Software Suite. Trends Genet 16: 276–277. 10827456 10.1016/s0168-9525(00)02024-2

[26] Kobayashi S, Ji H, Yuza Y, Meyerson M, Wong KK, et al (2005) An alternative inhibitor overcomes resistance caused by a mutation of the epidermal growth factor receptor. Cancer Res 65: 7096–7101. 16103058 10.1158/0008-5472.CAN-05-1346

[27] Mukohara T, Engelman JA, Hanna NH, Yeap BY, Kobayashi S, et al (2005) Differential effects of gefitinib and cetuximab on non-small-cell lung cancers bearing epidermal growth factor receptor mutations. J Natl Cancer Inst 97: 1185–1194. 16106023 10.1093/jnci/dji238

