# Supplementary material for: HeurAA: Accurate and Fast Detection of Genetic Variations with a Novel Heuristic Amplicon Aligner Program for Next Generation Sequencing
Source: PLoS One. 2013 Jan 18;8(1):e54294. doi: 10.1371/journal.pone.0054294 (PMC3548894; doi:10.1371/journal.pone.0054294)
Supplement: File S1 — Reference sequences. Reference sequences used for accuracy and speed comparison. (DOCX) [file pone.0054294.s001.docx]

PLOS One (Supplementary material)

*HeurAA*: Accurate and fast detection of genetic variations with a novel heuristic amplicon aligner program for next generation sequencing

Lőrinc S. Pongor, Ferenc Pintér and István Peták

S1. Reference sequences. Reference sequences used for accuracy and speed comparison.

S1. Reference Sequences.

>Epidermal growth factor receptor exon 19, 4831698 – 4831982 bp segment

CTGGTAACATCCACCCAGATCACTGGGCAGCATGTGGCACCATCTCACAATTGCCAGTTAACGTCTTCCTTCTCTCTCTGTCATAGGGACTCTGGATCCCAGAAGGTGAGAAAGTTAAAATTCCCGTCGCTATCAAGGAATTAAGAGAAGCAACATCTCCGAAAGCCAACAAGGAAATCCTCGATGTGAGTTTCTGCTTTGCTGTGTGGGGGTCCATGGCTCTGAACCTCAGGCCCACCTTTTCTCATGTCTGGCAGCTGCTCTGCTCTAGACCCTGCTCATCTC

>Epidermal growth factor receptor exon 20, 4838312 – 4838535 bp segment

CCACCATGCGAAGCCACACTGACGTGCCTCTCCCTCCCTCCAGGAAGCCTACGTGATGGCCAGCGTGGACAACCCCCACGTGTGCCGCCTGCTGGGCATCTGCCTCACCTCCACCGTGCAGCTCATCACGCAGCTCATGCCCTTCGGCTGCCTCCTGGACTATGTCCGGGAACACAAAGACAATATTGGCTCCCAGTACCTGCTCAACTGGTGTGTGCAGATCG

**>**Phosphatidylinositol-4,5-bisphosphate 3-kinas, 85431149 – 85431348 bp segment

CAGACTAGCTAGAGACAATGAATTAAGGGAAAATGACAAAGAACAGCTCAAAGCAATTTCTACACGAGATCCTCTCTCTGAAATCACTGAGCAGGAGAAAGATTTTCTATGGAGTCACAGGTAAGTGCTAAAATGGAGATTCTCTGTTTCTTTTTCTTTATTACAGAAAAAATAACTGAATTTGGCTGATCTCAGCATGT

>PRP_OCTA reference sequence Human Major Prion Protein (NT_011387/ENSG00000171867) octapeptide repeat region, exon 2 (first coding exon) 4620050 - 4620222 bp segment, exon starts: -122

GGGCAGCCCTGGAGGCAACCGCTACCCACCTCAGGGCGGTGGTGGCTGGGGGCAGCCTCATGGTGGTGGCTGGGGGCAGCCCCATGGTGGTGGCTGGGGACAGCCTCATGGTGGTGGCTGGGGTCAAGGAGGTGGCACCCACAGTCAGTGGAACAAGCCGAGTAAGCCAAAAACCAACATGAAGCACATGGCTGGTGCTGCAGCAGCTGGACGTAACGTAA
